# Exploring the Medicinal Potential of *Achillea grandifolia* in Greek Wild-Growing Populations: Characterization of Volatile Compounds, Anti-Inflammatory and Antioxidant Activities of Leaves and Inflorescences

**DOI:** 10.3390/plants12030613

**Published:** 2023-01-30

**Authors:** Olga S. Tsiftsoglou, Maria-Eleni Atskakani, Nikos Krigas, Michalis K. Stefanakis, Christos Gounaris, Dimitra Hadjipavlou-Litina, Diamanto Lazari

**Affiliations:** 1 Laboratory of Pharmacognosy, School of Pharmacy, Aristotle University of Thessaloniki, 54124 Thessaloniki, Greece; 2 Institute of Plant Breeding and Genetic Resources, Hellenic Agricultural Organization Demeter, 57001 Thessaloniki, Greece; 3 Department of Chemistry, University of Crete, 71003 Heraklion, Greece; 4 Department of Pharmaceutical Chemistry, School of Pharmacy, Faculty of Health Sciences, Aristotle University of Thessaloniki, 54124 Thessaloniki, Greece

**Keywords:** Asteraceae, essential oils, GC-MS, thujone bearing plants, LOX, DPPH

## Abstract

Various species of the genus *Achillea* L. (Asteraceae) are traditionally used worldwide for wound healing against diarrhea, flatulence, and abdominal pains, as diuretic and emmenagogue agents. In the present study, the essential oils (EOs) obtained separately from the leaves and inflorescences of wild-growing *Achillea grandifolia* Friv. from Mt. Menoikio and Mt. Pelion (Greece) were analyzed by Gas Chromatography–Mass Spectrometry. The major compounds found in EOs of *A. grandifolia* inflorescences from Mt. Menoikio were as follows: *cis*-thujone (36.9%), 1,8-cineole (11.9%), camphor (10.0%), ascaridole (7.3%), α-terpinene (6.4%), sabinene (4.1%), *trans*-thujone (3.6%), and *cis*-jasmone (3.4%). In leaves from Mt. Menoikio, they were as follows: *cis*-thujone (50.8%), 1,8-cineole (20.0%), *trans*-thujone (5.5%), camphor (5.5%), borneol (3.6%), and α-terpineol (3.1%). In inflorescences from Mt. Pelion, they were as follows: camphor (70.5%), camphene (5.9%), *cis*-jasmone (3.2%), bornyl acetate (3.2%). In leaves from Mt. Pelion, they were as follows: camphor (83.2%), camphene (3.9%), and borneol (3.7%). Subsequently, the samples were first time tested for their antioxidant activities with the interaction of EOs with DPPH (2,2-diphenyl-1-picrylhydrazyl) and their inhibition of lipid peroxidation, as well as for their anti-inflammatory activity through the soybean LOX (lipoxygenase) inhibition. All of the examined samples were found effective. *A. grandifolia* leaves presented the highest antioxidant potential according to the DPPH method, and the highest percentage of LOX inhibition. The study herein investigated for the first time the leaves and the inflorescences of *A. grandifolia* separately, and the results generally align with similar studies from neighboring countries (Turkey and Serbia) in terms of the yields and categorization of main EO compounds (oxygenated monoterpenes). However, the findings were not in agreement with previously studied Greek material, as a higher amount of *cis*-thujone and lower antioxidant activity are reported herein. Both the EOs of inflorescences and the leaves of the wild-growing population collected from Mt. Menoikio were characterized by a high quantity of *cis*-thujone (36.9% and 50.8%, respectively).

## 1. Introduction

The genus *Achillea* L. (Asteraceae) includes about 130 species worldwide [1]. The name *Achillea* has its roots in Greek mythology, originating from the ancient Greek hero Achilles, who is believed to have used yarrow to treat his soldiers’ wounds during the Trojan War [2]. The most common species in this genus is *A. millefolium* L., which is commonly known as “yarrow” and has been used for thousands of years in folk medicine due to its pharmaceutical properties such as anti-inflammatory of hepato-biliary and an anti-spasmodic effect on gastro-intestinal disorders [3]. The genus *Achillea* in Greece comprises about 25% of the genus’ diversity, namely 32 species and subspecies including 7 Greek endemic ones [4]. Today, many *Achillea* spp. have been traditionally used in wound healing, for abdominal pain, against diarrhea and flatulence, for symptomatic relief of colds and ulcer, as diuretic or emmenagogue, appetizer, carminative, and as insecticidal agents [5,6,7,8,9]. Besides their scientifically approved medicinal applications such as anti-inflammatory, antioxidant, anticancer, spasmolytic, and choleretic, *Achillea* spp. Are also used as additives in food products, in gardening as ornamentals, or for cut flowers, while their essential oils and extracts are also used in cosmetology [10,11].

*Achillea grandifolia* Friv. belongs to the Sectio *Millefolium* (DC.) W. Koch, together with the closely related and widespread *A. millefolium.* In contrast with the latter, *A. grandifolia* is a range-restricted species which is endemic to the south and central parts of the Balkan Peninsula extending to Anatolia in Turkey. Within this range, it is naturally found as a wild-growing native plant in woodlands and forest edges, from intermediate altitudes and forested mountainous areas to subalpine levels [12,13]. To date, literature data reporting the composition of the essential oils extracted from aerial parts of *A. grandifolia* are limited. All previous studies have investigated plant material from wild-growing populations [14,15,16,17,18,19,20,21], as well as from cultivated plants in Kazakhstan [22], and most of the extant information is related with the wild populations of Turkey [20,21] or Serbia [15,16,17,18,19]. There is only one study concerning the essential oil of wild-growing *A. grandifolia* from Greece, originating from the deciduous *Carpinus* forest of Vikos Gorge (Pindus Mountain range, Epirus) at an altitude of 500 m [14].

In this framework, the aim of this study was firstly to investigate the essential oil composition in the leaves and the inflorescences of the wild-growing populations of *A. grandifolia* found in Greece, and, consequently, to explore their respective antioxidant potential and anti-inflammatory activities. It must be mentioned that to the best of our knowledge there are no other reported studies concerning the anti-inflammatory activity of the essential oil of this particular *Achillea* species. The study herein is also the first one investigating separately the essential oils in the inflorescences and the leaves of this species; this is actually in line with similar investigations that have already been performed previously in other members of *Achillea* such as *A. ligustica* All. [23] and *A. coarctata* Poir. From Greece [24]. The investigation herein is aimed to update and amend an old phytochemical study that examined plant material originating from Greece [14], and to complement or contrast with other similar studies from Serbia [15,16,17,18,19] and Turkey [20,21].

## 2. Results and Discussion

### 2.1. Composition of the Studied Essential Oils

**Figure 1:**
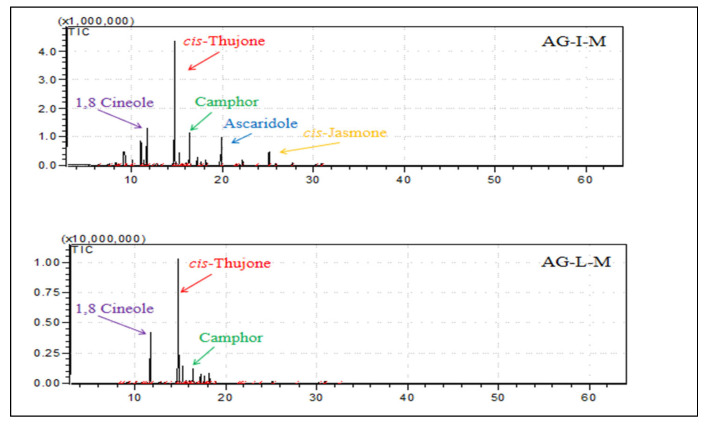
GC-MS chromatographs from the analysis of the essential oils of *Achillea grandifolia* collected from Mt. Menoikio, Greece (AG-I-M: Inflorescence; AG-L-M: Leaves) during full flowering (July 2020).

**Figure 2:**
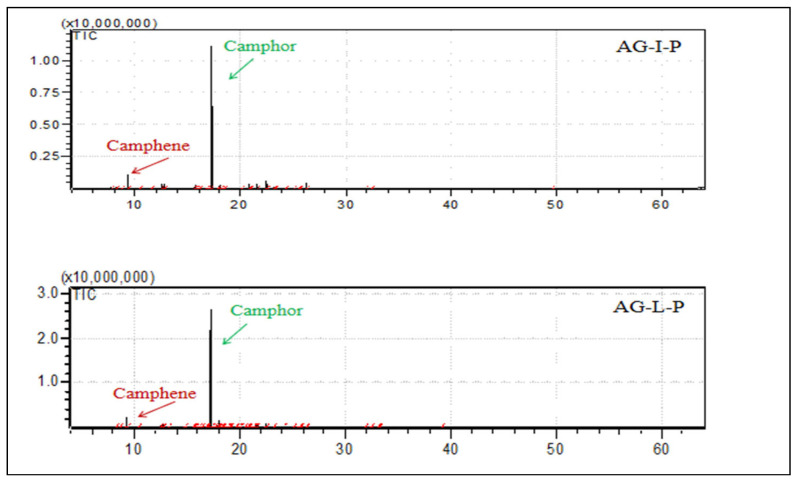
GC-MS chromatographs from the analysis of the essential oils of *Achillea grandifolia* collected from Pelion, Greece (AG-I-P: Inflorescence; AG-L-P: Leaves) during full flowering (July 2019).

The more complex essential oils were those obtained from Mt. Menοikio inflorescences (AG-I-M) with 30 compounds, followed by the corresponding sample of leaves from the same area (AG-L-M) with 27 compounds, whereas Mt. Pelion’s inflorescences (AG-I-P) and leaves (AG-L-P) were quite simpler (11 and 6 compounds, respectively) (Figure 1 and Figure 2). It is notable that the relative compositions of the studied samples of essential oils were totally different. Despite the detected differences, however, the majority of the components were oxygenated monoterpenes while the essential oil obtained from the inflorescences in both cases (samples from Mt. Menoikio and Mt. Pelion) was almost 10% richer in detected compounds than that of the leaves. It should be noted that none of the two studied essential oils (leaves, inflorescences) from Mt. Pelion provided sesquiterpenoids. Moreover, the essential oils of the inflorescences were richer in monoterpenoids hydrocarbons compared to leaves from the same wild-growing populations. Furthermore, *p*-cymene and *cis*-jasmone were found in higher quantity in essential oil from inflorescences compared with that of the leaves. The proportion of α-terpinene and ascaridole in the essential oil from the inflorescences of Mt. Menoikio (AG-I-M) was high, and characterized only this particular sample. Previous phytochemical studies in *A. grandifolia* from Greece have reported that the most abundant compounds in its essential oil are camphor (25.6%), 1,8-cineole (12.8%), *cis*-thujone (11.9%), and *trans*-thujone (9.2%) [14]. However, these compounds and their proportions differ considerably from those reported in the present study.

Table 1 presents the yields of the volatile fractions separately for leaves and inflorescences, the percentage (%) of identified compounds, as well as major group compounds of the studied samples. All the different components (n = 38) of the essential oils of the studied samples are listed in Table 1 according to their increasing retention times. In all cases, the extracted oil was yellowish to green in color. The yields of the volatile fractions obtained from *A. grandifolia* from Mt. Pelion’s inflorescences (AG-I-P) and leaves (AG-L-P) were 0.38% and 0.12%, respectively, while those from Mt. Menoikio (AG-I-M and AG-L-M) were 0.25% and 0.16%, respectively, on dry weight basis.

Regarding *A. grandifolia* from Mt. Pelion, at least 11 compounds were identified in the essential oil from its inflorescences (representing 91.4% of the total oil extracted), and 7 compounds were identified in the essential oil of its leaves (representing 95.5% of the oil extracted), while 6 compounds were found both in its inflorescences and leaves. Both of these essential oils were classified as camphor chemotype. Concerning *A. grandifolia* from Mt. Menikio, at least 30 compounds were identified in the essential oil from its inflorescences (representing 97.9% of the total oil extracted) and 27 compounds were identified in the EO of its leaves (representing 97% of the oil extracted), while 23 compounds were found both in its inflorescences and leaves. Both of these essential oils were classified as *cis*-thujone chemotype.

### 2.2. Biological Activities

In this study, the essential oils were evaluated in vitro for their antioxidant activities through their anti-lipid peroxidation activities and their interaction with the free stable radical DPPH, as well as for their anti-inflammatory activity through the in vitro inhibition of soybean lipoxygenase (LOX). Lipoxygenase (LOX) is the key enzyme involved in membrane lipid peroxidation by forming hydroperoxides, thus it is considered a target for inflammatory diseases. LOX inhibitors may act either as radical scavengers or inhibitors of free radical production, since lipoxygenation occurs via a carbon-centered radical. LOX inhibitors bearing an antioxidant profile could be expected to offer protection in inflammatory conditions and lead to potentially effective drugs. For the in vitro study, the soybean lipoxygenase was used due to a homology with mammalian lipoxygenase. All the tested essential oils of *A. grandifolia* inhibited the LOX, and the results are given in Table 2. None of the examined samples was found to be a better inhibitor than the NDGA which was used as a reference compound. This experiment describes for the first time the anti-inflammatory activity of the studied essential oils. Despite the complex chemical differences reported herein, it seemed that the essential oil derived from the leaves of the studied plant materials (AG-L-M and AG-L-P) was more active than that of inflorescences in both cases examined (AG-I-M and AG-I-P). A probable reason for this is the high content of oxygenated monoterpenoids in the essential oil of the studied leaves. Among the examined oils, only AG-I-M (inflorescences from Mt. Menoikio) presented anti-lipid peroxidation, with 47% compared to TROLOX used as reference. This essential oil was the only one with sesquiterpenoids hydrocarbons (1.0%), and also was the one with high amounts of monterpenoid hydrocarbons (17%, two-fold more than the previous). In both cases of the essential oils extracted from inflorescences and leaves, their reducing ability was time dependent. Thus, it seemed that the antioxidant activity in the DPPH assay was increased only after an hour of interaction.

It is known that many parameters may influence the composition of the essential oils such as harvesting period, plant tissue, geographical and climate conditions, age and development stage, nutrition, post-harvest drying, etc. [25]. Such aspects are in accordance with the results of the current study reporting different essential oil profiles for different organs such as leaves and inflorescences.

The detection of ascaridole and *cis*-jasmone may provide support about the taxonomic identity of the plant material, since *A. grandifolia* is often misidentified in the wild by herb collectors as *Tanacetum macrophyllum* (Waldst. & Kit.) Sch. Bip. Despite the apparent external morphological similarities between the two species, there are significant phytochemical differences between the composition of their essential oils. The essential oil of *A. grandifolia* is characterized by *cis*-jasmone and ascaridole, coupled with low quantities of sesquiterpenoids; on the other hand, the essential oil of *T. microphyllum* is characterized by a high concentration of oxygenated sesquterpenoids [18].

Thujone is known to be toxic (neurotoxicant since 1800’s) [26], and, therefore, the long-term usage of thujone-containing plant materials such as cedar leaves or *Artemisia absinthium* L. or *Salvia officinalis* L. was prohibited in Europe, while in the USA such materials are not authorized for usage as flavoring substances (EMA/HMPC/732886/2010). Although their use has been permitted at least in Europe, the levels of thujone isomers have to be carefully controlled and should be kept under specified limits to be used. In an essential oil sample from Serbian plant material of *A. grandifolia*, *trans*-thujone (*β*-thujone), i.e., the less toxic of the two isomers, was one of the major constituents [18]. On the contrary, the herein presented results showed that *cis*-thujone (α-thujone), which is the most toxic isomer, was among the main compounds in the plant material examined from Mt. Menoikio.

Generally, the major category of compounds to be detected in the essential oil of every *Achillea* species is expected to be that of monoterpenoids (both hydrocarbons and oxygenated) [27]. In all of the essential oils of *Achillea* spp. extracted from plant material collected from Greece to date, the majority of monoterpenoids are reported to be oxygenated, and in almost every studied sample among them, camphor and 1,8-cineole are reported as prevailing compounds [14,23,24,28,29,30]. This general trend seems to be in accordance with the chemical profile of the essential oil of *A. grandifolia*, collected both from Mt. Pelion and from Mt. Menoikio. It must be mentioned that there are two studies revealing that the main compound of some *Achillea* species collected from Greece is that of sesquiterpenes, such as ascaridole (47%) in *A. milefollium* L. from North Greece [31] and α-bisabolol (53.88%) in *A. cretica* L. from Crete [32]. Both the essential oils of the inflorescences and leaves of the wild-growing population collected from Mt. Menoikio were characterized by a high quantity of *cis*-thujone (50.8% in leaves and 36.9% in inflorescence). There is no other study reporting such a high amount of *cis*-thujone and *trans*-thujone in *A. grandifolia* essential oils. Previous phytochemical analysis of *A. grandifolia* essential oil from Serbia [15,16,17,18] and Greece [14] also report that *cis*-thujone (α-thujone) is among the main compounds, but in moderate quantities, i.e., 7.5%, 14.0%, and 11.9%, respectively. Furthermore, none of the samples examined from wild-growing plant material collected in Turkey (n = 3) has been reported to detect thujone in noteworthy quantities, except some traces (amounts not more than 0.05%) found in a sample from Aydin province [21].

The antiradical capacity of the essential oils extracted from all aerial parts of *A. grandifolia* has been previously reported [16,17,19,20]. In previous studies, all the examined essential oils of *A. grandifolia* have been tested for antioxidant activity using in vitro (non-enzymatic systems employing different model substrates) methods such as 2,2- diphenyl-1-picrylhydrazyl (DPPH) radical scavenging and Trolox equivalent antioxidant capacity tests (cation radical ABTS^+•^). The essential oil originating from Serbian plant material [19] seems to exhibit concentration-dependent antiradical activity, with a SC_50_ value at 6 µL/mL (DPPH). Moreover, this oil has also been tested using the TLC-DPPH assay, revealing a few light-yellow spots, which indicate that some of the components exhibit anti-DPPH activity. The same methods were also performed in other studies [16], and the results were almost the same as previously mentioned (SC_50_ value of 5.4 mg/mL and TLC-DPPH assay, revealing two main light-yellow spots, which indicate the anti-DPPH activity of 1,8-cineole and camphor). In other studies, the essential oil is reported to exhibit free radical scavenging activity against the DPPH^•^ radicals (52%) [17,20] and the ABTS^+•^ cation radicals (TEAC 0.62 mM) [20], or moderate Trolox-equivalent antioxidant capacity (0.62 mM) against ABTS [17]. The results of the antioxidant activity assays herein showed that the oil of *A. grandifolia* could be considered as a source of effective antioxidants, indicating a moderate antioxidant activity which was lower compared to previous literature data.

A limited number of studies concerning the anti-inflammatory activity of essential oil extracted from *Achillea* spp. [33,34] has been published to date. Most of the published research has investigated the anti-inflammatory activity of the polar extracts from *Achillea* spp., especially extracts from *A. millefolium*, using in vivo techniques [35,36]. This is the first report on the anti-inflammatory activity of the essential oil of *A. grandifolia*. Specifically, AG-I-M presented the stronger anti-lipid peroxidation with 47%, compared to TROLOX used as a reference compound. None of the other essential oils exhibited any activity. These results are possibly related to the different composition of this essential oil, since it was the only one containing more than 10% of monoterpene hydrocarbons (17%), sesquiterpene hydrocarbons (1%), as well as ascaridole (7.5%).

## 3. Materials and Methods

### 3.1. Collection of Plant Material

**Figure 3:**
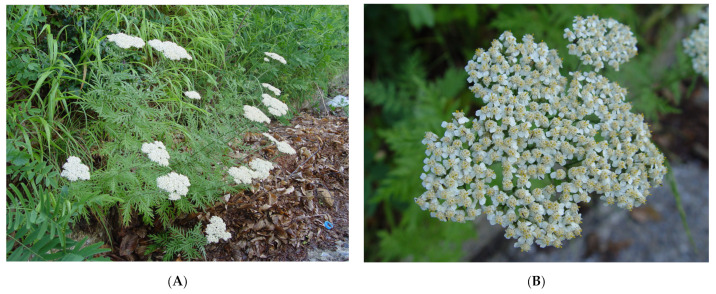
Leafy above-ground parts (**A**) in flower, and vertical view of the compound inflorescences (**B**) of *Achillea grandifolia* in its original habitat on Mt. Pelion (Greece).

The botanical collections were performed using a special collection permit issued by the Greek Ministry of Environment (182336/879 of 16-5-2019 and 64886/2959 of 6-7-2020). Above-ground parts of wild-growing populations of *A. grandifolia* (Figure 3) were collected during summer 2020 from Mountain Menoikio (Eastern Macedonia, Greece; Longitude: 23°42′32.77″ E, Latitude: 41°10′33.81″ N), and during summer 2019 from Mountain Pelion (Southeastern Thessaly, Greece; Latitude: 39°26′18.84″ N, Longitude: 23°02′46.57″ E). The original collection of samples in the wild comprised about 300 g of fresh above-ground plant material carefully excised with scissors to allow vegetative plant regeneration. The plant material was separated into leaves and inflorescences before drying. The collected materials were spotted in situ and later taxonomically identified by Dr. N. Krigas (experienced plant taxonomist), and voucher specimens from each locality (Lazari D. 7512 and No Lazari D. 7347) were deposited at the School of Pharmacy, Aristotle University of Thessaloniki (Greece).

### 3.2. Essential Oil Isolation

The collected plant material was air-dried at room temperature in dark and shade for ten days. Each sample was submitted three times to hydrodistillation for two h, using a modified Clevenger-type apparatus with a water-cooled oil receiver to reduce hydrodistillation overheating artifacts. The volatiles were trapped in 5 mL gas chromatography grade n-pentane according to standard procedures [37], were dried over anhydrous sodium sulfate, and were kept in closed, air-tight Pyrex containers at −4 °C. The essential oil yield was expressed as mL per 100 g d.w.

### 3.3. Gas Chromatography-Mass Spectrometry

The essential oil analyses were performed on a Shimadzu GC-2010-GCMS-QP2010 (Shimadzu, Kyoto, Japan) system operating at 70 eV. This was equipped with a split/splitless injector (230 °C) and a fused silica HP-5 MS capillary column (30 m × 0.25 mm i.d., film thickness 0.25 μm). The temperature program was from 50 °C to 290 °C, at a rate of 4 °C/min. Helium was used as a carrier gas at a flow rate of 1.0 mL/min. The injection volume of each sample was 1.0 μL. Retention indices for all compounds were determined according to Van den Dool and Kratz [38], using n-alkanes as standards. The identification of the components was based on comparison of their mass spectra with those of NIST21 and NIST107 [39], and by comparison of their retention indices with literature data [40]. The studied essential oils were subjected to co-chromatography with authentic compounds (Fluka, Sigma, Burlington, MA, USA).

### 3.4. In Vitro Experiments

For the in vitro experiments, stock solutions of the studied essential oils containing 20 µL/100 µL of absolute ethanol were used, from which the appropriate samples were taken and used in the following assays. All the volumes used are indicated in Table 2, and reference compounds as 10 mM stock solutions in DMSO were also used (Table 2).

#### 3.4.1. Inhibition of Linoleic Acid Lipid Peroxidation

For the inhibition of linoleic acid lipid peroxidation, the method of Hadjipavlou and Pontiki [41] was followed as described by Hodaj-Çelikuet al. [42]. In brief, the tested samples were dissolved in ethanol, and a stock solution containing 20 µL/100 µL in ethanol was prepared. The production of conjugated diene hydroperoxide by oxidation of linoleic acid in an aqueous dispersion was monitored at 234 nm in the presence of 2,2′-Azobis (2-amidinopropane) dihydrochloride (AAPH) of 50 μL of 40 mM AAPH solution as a free radical initiator in 0.05 M phosphate buffer, pH 7.4. Oxidation was carried out in the presence of the tested samples (10 μL from the stock solution). The rate of oxidation at 30 °C room temperature was monitored by recording the increase in absorption at 234 nm caused by conjugated diene hydroperoxides. Trolox was used as a reference drug. For the calculation of the in vitro antioxidant assays, the formula (A_0_ – A_1_)/A_0_ × 100 was used, where A_0_ is the control absorbance and A_1_ is the sample’s absorbance.

#### 3.4.2. Soybean Lipoxygenase Inhibition Study In Vitro

This analysis was performed according to the assay developed by Peperidou et al., [43] using 10 μL from a stock solution of 20 µL/100µL of absolute ethanol. Each of the samples was incubated at room temperature with sodium linoleate (0.1 mM) and 0.2 mL of enzyme solution (1/9 × 10^−4^ *w*/*v* in saline) in tris buffer pH 9. The conversion of sodium linoleate to 13-hydroperoxylinoleic acid was recorded at 234 nm and the percentage of inhibition resulted from essential oil was compared with the appropriate standard inhibitor nordihydroguaretic acid (NDGA). For the calculation of the in vitro LOX inhibition, the formula (A_0_ − A_1_)/A_0_ × 100 was used, where A_0_ is the control absorbance and A_1_ is the sample’s absorbance.

#### 3.4.3. Interaction with DPPH

The tested samples were dissolved to a 10 μL solution of DPPH (0.1 mM in methanol), and 20 µL/100 µL ethanol stock solution was added after 20-60 min. The antioxidant activity was recorded at 517 nm and the percentage of reducing activity (RA) was calculated and compared to the reference compound NDGA (nordihydroguaiaretic acid). The method followed was according to the description of Peperidou et al. [43]. For the calculation of the in vitro antioxidant assays, the formula (A_0_ − A_1_)/A_0_ × 100 was used, where A_0_ is the control absorbance and A_1_ is the sample’s absorbance.

## 4. Conclusions

The study herein reports worldwide for the first time the essential oil’s composition in leaves and inflorescences of *A. grandifolia* examined separately and not as above-grounds parts. This research provides detailed insight regarding the chemical compounds (n = 38) of the essential oils in *A*. *grandifolia* plant material originating from Greece, aligning with similar studies from neighboring countries (Turkey and Serbia) in terms of yields, color, and smell, as well as the categorization of the main compounds of the essential oil (oxygenated monoterpenes). All examined essential oils were found effective in terms of antioxidant potential and anti-inflammatory activity, the latter reported for the first time herein. However, the findings are not in agreement with previous studies.

The analysis of the essential oils suggested the existence of distinct chemotypes of *A. grandifolia* in Greece, i.e., camphor chemotype from Mt. Pelion and *cis*-thujone chemotype from Mt. Menoikio, with the latter major compound being reported for the first time herein as prevailing both in leaves (almost 51%) and inflorescences (almost 37%). The results of the antioxidant activity assays herein showed that the oil of *A. grandifolia* could be considered as a source of effective antioxidants, and actually indicated a moderate antioxidant activity which was lower compared to previous literature data.

## Figures and Tables

**Table 1:** Composition of the essential oils of *Achillea grandifolia* (AG) inflorescences (-I) and leaves (-L) collected from Greek wild-growing populations during full flowering from Mt. Menoikio (-M) in July 2020 and Mt. Pelion (-P) in July 2019.

Mountain						Mt. Menoikio (-M)		Mt. Pelion (-P)		
Percentage of Yield						0.25%	0.16%	0.38%	0.12%	Retention Times
	^a^ Compounds	^b^ MF	^c^ t_R_	^d^ RI_exp_	^e^ RI_lit_	AG-I-M	AG-L-M	AG-I-P	AG-L-P	
1	Santolina triene	C_10_H_16_	6.454	903	906	0.2 ± 0.01		nd	nd	RI, MS
2	Tricyclene	C_10_H_16_	7.101	920	921	nd	nd	0.3 ± 0.01	nd	RI, MS
3	*α*-Pinene	C_10_H_16_	7.621	931	932	0.5 ± 0.01	nd	0.2 ± 0.00	nd	RI, MS Co-GC
4	Camphene	C_10_H_16_	8.287	948	946	0.8 ± 0.01	0.1 ± 0.01	5.9 ± 0.14	3.9 ± 0.01	RI, MS
5	Sabinene	C_10_H_16_	9.257	971	969	4.1 ± 0.27	0.3 ± 0.01	nd	nd	RI, MS
6	*β*-Pinene	C_10_H_16_	9.417	975	974	0.2 ± 0.00	0.1 ± 0.00	0.1 ± 0.01	nd	RI, MS, Co-GC
7	Dehydro-1,8-Cineole	C_10_H_16_O	9.983	989	988	nd	0.1 ± 0.00	nd	nd	RI, MS
8	*δ*-2-Carene	C_10_H_16_	10.160	994	1001	1.5 ± 0.01	0.1 ± 0.01	nd	nd	RI, MS
9	Hexyl acetate	C_8_H_16_O_2_	10.752	1008	1007	nd	0.2 ± 0.00	nd	nd	RI, MS
10	*α*-Terpinene	C_10_H_16_	11.084	1016	1014	6.4 ± 0.19	0.1 ± 0.01	nd	nd	RI, MS
11	*p*-Cymene	C_10_H_14_	11.442	1025	1022	1.6 ± 0.07	0.4 ± 0.01	2.00 ± 0.11	0.4 ± 0.03	RI, MS, Co-GC
12	1,8-Cineole	C_10_H_18_O	11.704	1032	1026	11.9 ± 0.52	20.0 ± 0.62	2.2 ± 0.07	1.3 ± 0.06	RI, MS, Co-GC
13	*trans*-β-Ocimene	C_10_H_16_	12.384	1048	1044	0.2 ± 0.01	nd	nd	nd	RI, MS
14	*γ*-Terpinene	C_10_H_16_	12.809	1059	1054	0.3 ± 0.01	0.4 ± 0.01	nd	nd	RI, MS, Co-GC
15	*cis*-Sabinene hydrate	C_10_H_18_O	13.339	1072	1065	0.2 ± 0.01	0.4 ± 0.17	nd	nd	RI, MS
16	Artemisia alcohol	C_10_H_18_O	13.735	1081	1080	0.5 ± 0.00	nd	nd	nd	RI, MS
17	Terpinolene	C_10_H_16_	13.879	1085	1086	0.2 ± 0.01	0.1 ± 0.00	nd	nd	RI, MS
18	Linalool	C_10_H_18_O	14.591	1102	1095	0.6 ± 0.01	0.7 ± 0.01	nd	nd	RI, M, Co-GC MS
19	*cis*-Thujone	C_10_H_16_O	14.816	1108	1101	36.9 ± 0.93	50.8 ± 1.45	1.5 ± 0.04	nd	RI, MS
20	*trans*-Thujone	C_10_H_16_O	15.253	1120	1112	3.6 ± 0.03	5.5 ± 0.11	0.1 ± 0.01	nd	RI, MS
21	*cis*-p-Menth-2-en-1-ol	C_10_H_18_O	15.492	1126	1118	0.1 ± 0.00	0.2 ± 0.01	nd	nd	RI, MS
22	*trans*-Sabinol	C_10_H_16_O	16.089	1141	1137	0.9 ± 0.02	1.1 ± 0.05	nd	nd	RI, MS
23	Camphor	C_10_H_16_O	16.376	1149	1141	10.0 ± 0.27	5.5 ± 0.08	70.5 ± 1.07	83.2 ± 0.85	RI, MS
24	*trans*-Verbenol	C_10_H_16_O	16.652	1145	1140	nd	nd	nd	1.20± 0.01	RI, MS
25	*cis*-Chrysanthenol	C_10_H_16_O	16.991	1165	1160	nd	0.2 ± 0.01	nd	nd	RI, MS
26	Borneol	C_10_H_18_O	17.317	1173	1165	2.4 ± 0.51	3.6 ± 0.21	2.2 ± 0.18	3.7 ± 0.24	RI, MS, Co-GC
27	Terpinen-4-ol	C_10_H_18_O	17.651	1182	1174	0.9 ± 0.01	2.2 ± 0.14	nd	nd	RI, MS, Co-GC
28	*α*-Terpineol	C_10_H_18_O	18.244	1197	1186	1.2 ± 0.03	3.1 ± 0.02	nd	nd	RI, MS, Co-GC
29	*trans*-Piperitol	C_10_H_18_O	18.757	1212	1207	nd	0.1 ± 0.01	nd	nd	RI, MS
30	Ascaridole	C_10_H_16_O_2_	19.870	1242	1234	7.3 ± 0.08	nd	nd	nd	RI, MS
31	Bornyl acetate	C_12_H_20_O_2_	21.408	1285	1287	nd	nd	3.2 ± 0.04	nd	RI, MS
32	Eugenol	C_10_H_12_O_2_	23.806	1356	1356	0.2 ± 0.01	0.1 ± 0.00	nd	nd	RI, MS
33	*cis*-Jasmone	C_11_H_16_O	25.173	1396	1392	3.4 ± 0.15	0.7 ± 0.01	3.2 ± 0.33	1.8 ± 0.02	RI, MS
34	Methyl eugenol	C_11_H_14_O_2_	25.473	1405	1403	tr	nd	nd	nd	RI, MS
35	*β*-Caryophyllene	C_15_H_24_	25.867	1418	1417	0.4 ± 0.00	nd	nd	nd	RI, MS, Co-GC
36	10-epi-β-Acoradiene	C_15_H_24_	27.765	1478	1474	0.6 ± 0.02	nd	nd	nd	RI, MS
37	*trans*-Nerolidol	C_15_H_26_O	30.351	1563	1561	0.3 ± 0.01	0.1 ± 0.01	nd	nd	RI, MS
38	Caryophyllene oxide	C_15_H_24_O	30.954	1583	1582	0.5 ± 0.02	0.8 ± 0.09	nd	nd	RI, MS, Co-GC
**Total %**						**97.9**	**97.0**	**91.4**	**95.5**	
Monoterpene Hydrocarbons						17.0	1.6	8.5	4.30	
Oxygenated Monoterpenes						76.7	93.6	79.7	89.4	
Sesquiterpene Hydrocarbons						1.0	nd	nd	nd	
Oxygenated Sesquiterpenes						0.8	0.9	nd	nd	

^a^ Compounds listed in order of elution from an HP-5 MS capillary column; ^b^ MF: molecular formula; ^c^ t_R_: Retention time (min); ^d^ RI_exp_: Retention indices as determined on a HP-5 MS capillary column using a homologous series of n-alkanes (C9-C25); ^e^ RI_lit_: Retention indices according to literature; RI: Retention Index: MS: Mass Spectrum: Co-GC: co-injection with authentic compound; nd = not detected; tr: traces.

**Table 2:** Percentage (%) interaction of the essential oils of *Achillea grandifolia* (AG) inflorescences (-I) and leaves (-L) collected from Greek wild-growing populations during full flowering from Mt. Menoikio (-M) in July 2020 and Mt. Pelion (-P) in July 2019 with DPPH, their soybean LOX inhibitory activity (%), and their inhibition of lipid peroxidation (%).

Results	Interaction (%) with the Stable Free Radical of DPPH		Inhibition of LOX (%)	Inhibition of Lipid Peroxidation (%)
Time	20 min	60 min		
Concentration	20 μL	20 μL	10 μL	10 μL
AG-I-M	11.0	14.9	30	47
AG-L-M	7.6	32.1	70	n.a.
AG-I-P	2	n.a	51	n.a.
AG-L-P	7	n.a	70	n.a.
NDGA	81.0	93.0	92.0	
TROLOX				96.0

Stock solution 10 mM in DMSO; n.a.: no activity found under the experimental conditions. The results are the mean of 3–6 measurements and the standard deviation (SD) was less than 10%.

## Data Availability

All data referred to or generated in this study are included in tables or figures and are available upon request.
